# Non-medical risk factors associated with postponing elective surgery: a prospective observational study

**DOI:** 10.1186/s12910-021-00660-0

**Published:** 2021-07-13

**Authors:** Julia Becker, Gerald Huschak, Hannes-Caspar Petzold, Volker Thieme, Sebastian Stehr, Sven Bercker

**Affiliations:** 1 Department of Anesthesiology and Intensive Care Medicine, University Hospital of Leipzig, Liebigstrasse 20, 04103 Leipzig, Germany; 2 Operating Room Management, University Hospital of Leipzig, Leipzig, Germany; 3 Clinical Ethics Committee, University Hospital of Leipzig, Leipzig, Germany

**Keywords:** Operation room management, Cancellation of surgery, Undertreatment

## Abstract

**Background:**

Operation room (OR) planning is a complex process, especially in large hospitals with high rates of unplanned emergency procedures. Postponing elective surgery in order to provide capacity for emergency operations is inevitable at times. Elderly patients, residents of nursing homes, women, patients with low socioeconomic status and ethnic minorities are at risk for undertreatment in other contexts, as suggested by reports in the medical literature. We hypothesized that specific patient groups could be at higher risk for having their elective surgery rescheduled for non-medical reasons.

**Methods:**

In this single center, prospective observational trial, we analysed 2519 patients undergoing elective surgery from October 2018 to May 2019. A 14-item questionnaire was handed out to illicit patient details. Additional characteristics were collected using electronic patient records. Information on the timely performance of the scheduled surgery was obtained using the OR’s patient data management system. 6.45% of all planned procedures analysed were postponed. Association of specific variables with postponement rates were analysed using the Mann–Whitney *U* test and Fisher's exact test/χ^2^-test.

**Results:**

Significantly higher rates of postponing elective surgery were found in elderly patients. No significant differences in postponing rates were found for the variables gender, nationality (Germany, EU, non-EU), native language, professional medical background and level of education. Significantly lower rescheduling rates were found in patients with ties to hospital staff and in patients with a private health insurer.

**Conclusions:**

Elderly patients, retirees and nursing home residents seem to be at higher risk for having their elective surgery rescheduled. However, owing to the study design, causality could not be proven. Our findings raise concern about possible undertreatment of these patient groups and provide data on short-term postponement of elective surgery.

*Trial registration* DRKS00015836. Retrospectively registered.

## Background

Operating room (OR) scheduling is a complex process that is influenced by multiple variables. Stringent planning can lead to optimized utilisation of costly resources. Reliability of scheduling is critical and can be increased by good preoperative evaluation and patient preparation. Multiple surgical theatres that are used on an interdisciplinary basis and with a high percentage of unplanned cases can complicate scheduling. Optimizing OR scheduling has been subject to numerous studies to improve on-site processes using methods of operations research [1]. This is not to be discussed further here, even if such considerations touch on aspects of a potential undersupply of patients.

In spite of all attempts to master stable OR scheduling many variables remain unpredictable and acute cancellation of a planned surgery slot commonly occurs. Reasons are medical as well as structural and are attributable partly to the patient, OR capacity, OR team performance or inadequate planning. Decision making in this context should be performed in an objective manner. It has, however, been shown, that cancellation due to non-medical reasons is common.

Life-threatening emergencies have the greatest potential for short-term harm, but relevant consequences of postponing an elective or semi-elective intervention have also been described. In cancer patients, an increase in mortality has been shown as a consequence of long-term delay of operations [2]. Recently, medical and psychological effects of postponing operations have been described extensively during the Covid-19 pandemic, e. g. an increase in anxiety in cancer patients [3] or an increase in impairment of physical function in patients awaiting to undergo hip arthroplasty [4]. A UK network analysed the psychological and economic burden induced by so-called “winter cancellations” in the NHS. Over one third of patients reported substantial sadness, disappointment, anger and stress and worsening of symptoms [5]. This mainly refers to long-term postponement. Substantial data on effects of short-term postponement have not been published yet. However, there are some obvious risks that may be associated with rescheduling operations (e.g. higher risk for high blood sugar levels in diabetic patients, reduced fluid intake leading to acute kidney injury)—prolonged phases of preoperative stress and anxiety with possible implications for long-term mental sequelae i.e. PTSD or depression.

We conducted this study to describe non-medical risk factors for cancellation of elective surgeries.

## Methods

This is an observational single-centre study situated at the University hospital of Leipzig. Between October 2018 and May 2019, all consecutive adult patients scheduled for elective surgery in the departments of 7 surgical subspecialties were screened during the preoperative anesthesia evaluation and asked to participate. The local ethics committee (Ref. No. 284/18) of the Medical Faculty of the University of Leipzig approved the study.

After informed consent was obtained, all patients were asked to complete a questionnaire including 14 items concerning age, gender, job (in particular if the patient had a professional medical background), place of birth, nationality, native language, living conditions (e.g. own flat or nursing home), kind of health insurance, if under legal supervision by a legal guardian, highest level of education and if patients had personal relationships to hospital staff. All questionnaires were available in German, English, French, Spanish, Turkish, Arab, Russian and Polish language.

Additional data were harvested using patient files and electronic OR schedules (SAP, SE Walldorf, Germany). As a routine procedure, every operation’s urgency is rated on a 5-point scale from “elective” (E) to “immediate operation without delay” (N0) as defined by the German societies of anaesthesiology, surgery and operating room management. For this study, only patients scheduled for elective surgery were analysed. This defines a planned elective surgical procedure without any urgency. To determine if an operation was postponed, daily schedules were automatically saved at midnight and then later compared with operations started during the next 24 h. If an elective surgery had had been scheduled on the OR plan at midnight and had not been started until the next 24 h, it was categorized as postponed. All postponed operations were checked for plausibility and evaluated if postponing was due to medical reasons (e.g. worsening clinical condition of the patient) or due to organizational issues (e.g. over-planning of the surgical capacities, postponement due to an emergency) or if patients cancelled the operations themselves.

For analysis, potentially confounding categories were grouped (e.g. age groups, origin from Germany, European Union (EU) or non-EU states, public or private health insurance). We hypothesized that non-EU patients scheduled for surgery were more likely to be postponed. Data analysis was performed using Mann–Whitney *U* test, Chi-Square test and Fisher´s exact test (statistical software package SPSS (v25, Siemens, Germany)). For age, residents of nursery homes and retired patients a logistic regression was performed to analyse independency of these variables.

## Results

During the study period n = 4396 elective patients were scheduled for operation. Of those, n = 2521 patients gave informed consent to participate in this study. 313 patients were excluded from further analysis due to several reasons. In n = 81 cases the scheduled surgery was not performed at all and in n = 188 inclusion criteria were violated (e. g. because priority changed from “elective” to a higher urgency category). 42 questionnaires could not be evaluated due to incorrect or missing information. As 365 patients had more than one operation during the study period, 2589 data records were analysed.

Of the 2589 scheduled patients that were analysed, 167 were postponed (6.45%). 2 patients cancelled the operation themselves and were excluded from further analysis. The mean age of all study participants was 54.9 years (SD 16.5 years). There were 52.1% male and 47.9% female patients. Data are shown in Table 1.

**Table 1:** Mean differences between groups. In brackets the distribution of the respective parameter within both groups

	Operation as scheduled (n = 2422)	Operation postponed (n = 165)	*p*-value	Odds ratio (OR) and 95% confidence interval (CI)
Age (years, mean, SD)	54.6 ± 16.5	59.4 ± 15.8	0.001	
Gender	52.1% (n = 1261) male 47.9% (n = 1161) female	52.1% (n = 86) male 47.9% (n = 79) female	1.000 n.s	OR: 1.002 [CI 0.731; 1.374]
Retirees	35.5% (n = 808) retirees	47% (n = 71) retirees	0.005	OR: 1.611 [CI 1.157; 2.243]
Country of birth in EU	96.8% (n = 2303) EU origin	96.9% (n = 157) EU origin	1.000 n.s	OR: 1.036 [CI 0.413; 2.598]
Nationality EU country	97.9% (n = 2347) EU nationality	98.8% (n = 159) EU nationality	0.463 n.s	OR: 1.694 [CI 0.408; 7.024]
Native language German	95.8% (n = 2277) native language German	97% (n = 159) native language German	0.684 n.s	OR: 1.411 [CI 0.567; 3.512]
Nursing home residents	0.58% (n = 14) nursing home residents	3% (n = 5) nursing home residents	< 0.001	OR: 5.348 [CI 1.903; 15.034]
Patients with private insurance	9.5% (n = 230) with private insurance	3.6% (n = 6) with private insurance	0.008	OR: 0.360 [CI 0.157; 0.822]
Under legal supervision	0.45% (n = 11) under legal supervision	2.4% (n = 4) under legal supervision	0.001	OR: 5446 [CI 1,715; 17,292]
Private relationship to staff	20.3% (n = 490) with relationship to staff	13.4% (n = 22) with relationship to staff	0.033	OR: 0.608 [CI 0.384; 0.963]
Professional medical background	9.8% (n = 237) with professional medical background	6.1% (n = 10) with professional medical background	0.132 n.s	OR: 0.593 [CI 0.308; 1.140]
Colonization with MDR pathogens	3.4% (n = 83) with MDR	10.3% (n = 17) with MDR	< 0.001	OR: 3.237 [CI 1.872; 5.597]
> 1 surgical intervention	28.3% (n = 686) with > 1 surgical intervention	37.6% (n = 62) with > 1 surgical intervention	0.013	OR: 1.523 [CI 1.098; 2.113]

Mann-Whitney U test, χ^2^-test/Fisher's exact test

Mean age was 54.9 years and patients with cancelled operations were significantly older (59.4 vs. 54.6 years). In patients aged > 79 years, the percentage of postponing was twice as high as in younger patients < 65 years of age (10.8% vs. 5.1%). Differences in postponing between the three age groups (< 65 y, 65–79 y, > 79 y) were significant (*p* = 0.001). In the univariate analysis, retirees (*p* = 0.005) and patients who lived in nursing homes (*p* < 0.001) had a significantly higher risk that elective surgery was not performed as scheduled. However, in the logistic regression only age turned out to be an independent risk factor.

Gender, origin or native language, and level of education did not have a significant effect on the incidence of postponing.

If patients had a personal relationship to staff members, the postponing rate was significantly lower (4.3% vs. 6.8%). The fact that patients had a professional medical background seemed to have no association with postponing. However, from the 48 physicians who were patients and participated in this study, only one case was postponed. Detailed results are shown in Table 1.

## Discussion

With the data presented here, we could show that postponing of elective surgery was significantly associated with non-medical factors. Elder, retired patients, residents of nursing homes and those colonized with multi-drug resistant (MDR) pathogens had an increased risk of an elective surgical case being postponed. The probability was significantly lower if patients had a private health insurance or had a personal relationship to hospital staff. In contrast to other studies origin, gender or being part of an ethnic minority was not associated with this effect. To our best knowledge, this is the first study addressing the association with non-medical factors with postponing elective surgery in a prospective, observational study.

The frequency of unplanned postponing was 8.9% in patients ≥ 65 years and only 5.1% in patients < 65 years. It is hardly possible to specify if this effect is due to procedural problems, to medical reasons or if this is an indicator for any kind of preventable undertreatment. In the medical literature, there are many examples showing undertreatment for elderly patients under specific circumstances. In a recent analysis, DuMontier et al. reviewed different studies describing undertreatment of elderly patients with different malignancies [6]. Undertreatment was defined as receiving a less intensive therapy than currently recommended.

Another example of undertreatment in elderly patients is the quality of pain treatment. Bernabei et al. demonstrated that elderly patients with cancer residing in nursing homes were more likely not to receive any pain medication or a reduced intensity of pain therapy, even though pain was very frequent in those patients. They postulated that underreporting and underestimation of pain in elderly patients may have contributed to this finding [7]. It could be possible that elderly patients suffering from dementia are more often incapable of expressing their needs and therefore more likely to be disadvantaged.

It is obvious that there must have been a substantial overlap between patients > 65 years, retirement and residents of nursing homes. Of all 879 retired patients 734 were > 64 years. On the other hand only 2 of 14 patients from nursing homes were younger than 65 years.

Numerous studies analysed reasons for cancellation of elective surgery. The main focus of these previous investigations lay on optimizing perioperative processes and patient preparation [8–12], and only three of these studies identified groups with an elevated risk of being cancelled. These retrospective studies demonstrated an association between cancellation and age, socioeconomic status and gender. Cho et al. analysed more than 60,000 surgical interventions in a Korean University hospital and found 8% cancellations with a significant correlation of cancellation with increasing age. An Iranian group analysed the effect of a health care transformation plan on surgical cancellations and showed an association between the type of insurance, marital status, sex and surgical cancellations [13]. However, there are many differences between the corresponding national health care systems and the reasons for these effects most likely differ substantially.

Nevertheless, aspects of health inequality and undertreatment in certain groups are well described in developed countries and age as a risk factor for inadequate treatment has been shown in several areas of medicine [14–16]. On the other hand, there is a multitude of medical reasons to provide less invasive treatment to elder patients with multi-system physiological changes and it is hard to distinguish between a rational decision and undertreatment. However, some examples of less intense or frequent therapy show aspects of undertreatment and it can be assumed to be the result of a kind of misconception of healthcare providers. In contrast, we hypothesize that the effects in our study are rather unwanted in nature and due to procedural problems or possibly due to psychological effects. We strongly believe that awareness concerning this phenomenon is of high importance to avoid undertreatment in patients at risk.

This study has some limitations. First of all, this is a single-centre study and we can only hypothesize that the effects are generalizable. However, the examples discussed above from the medical literature suggest that these effects are present or even widespread in health care systems of developed countries. As the reason for cancellation was analysed retrospectively and could not be proven in any case as non-medical in nature, we could not exclude that postponing was due to medical reasons at least in some cases. Second, this study was not designed to give a precise analysis of underlying reasons for surgical delay or analysed conceptions of the physicians responsible. Strictly speaking, the data provides an association—causality can be assumed but is not proven.

Furthermore, the low response rate might represent a bias. In general, groups with lower level of education or language barriers tend not to take part in surveys and we cannot exclude that those groups were underrepresented in our study.

This is a pre-pandemic study. It has been estimated that the current COVID-19 pandemic will lead to 28,404,603 cancellations of elective operations [17]. This shortage of medical resources has the potential to heighten the unequal treatment of patients. Future efforts should be directed towards better describing risk factors for inequality, identifying groups at risk, and developing programs to protect these groups from undertreatment.

## Conclusions

We demonstrated that elderly patients and nursing home residents planned for elective surgery have a higher incidence of case cancellation. We postulate that this association is due to a mixture of medical and non-medical reasons. These findings should lead to awareness towards possible undertreatment of vulnerable groups.

## Data Availability

The datasets used and analysed during the current study are available from the corresponding author on reasonable request.

## Abbreviations

OR: Operation room/Odds ratio
UK: United Kingdom
NHS: National Health Service
PTSD: Posttraumatic stress syndrome
SD: Standard deviation
MDR: Multi drug resistance
ICF: Informed consent form
